# Spatial distribution and incidence of bovine neonatal pancytopenia in Bavaria, Germany

**DOI:** 10.1186/s12917-020-02371-x

**Published:** 2020-05-24

**Authors:** Carola M. Sauter-Louis, Christoph Staubach, Frederike Reichmann, Alexander Stoll, Günter Rademacher, Klaus Cussler, Max Bastian, Annette Pfitzner-Friedrich

**Affiliations:** 1 Institute of Epidemiology, Friedrich-Loeffler-Institut, Suedufer 10, 17493 Greifswald, Isle of Riems Germany; 2 Clinic for Ruminants with Ambulatory and Herd Health Services at the Centre for Clinical Veterinary Medicine, LMU Munich, Sonnenstrasse 16, 85764 Oberschleissheim, Germany; 3 Paul-Ehrlich-Institut, Paul-Ehrlich-Straße 51-59, 63225 Langen, Germany; 4 Stikovet, Friedrich-Loeffler-Institut, Suedufer 10, 17493 Greifswald, Isle of Riems Germany

**Keywords:** BNP, Calf, Epidemiology, Haemorrhagic diathesis, BVD vaccine, Spatial, Bovine neonatal pancytopenia

## Abstract

**Background:**

Bovine neonatal pancytopenia (BNP) is a haemorrhagic disease of neonatal calves. BNP was first described in Germany in 2009, later on also in other European countries, and in New Zealand in 2011. The disease is characterised by spontaneous bleeding, pancytopaenia in the bone marrow, and a high case fatality ratio. The causal role of a specific bovine viral diarrhoea virus (BVDV) vaccine (PregSure®BVD, then Pfizer Animal Health, now Zoetis, Berlin, Germany) has been established over the last years, causing the production of alloantibodies in some vaccinated cattle, which in the case of pregnant cattle, are transferred to the newborn calf via the colostrum. However, striking regional differences in the incidence of the disease were observed within Germany and other countries, but as the disease was not notifiable, no representative data on the spatial distribution are available. In this study, we address the spatial distribution and incidence of BNP using the results of two representative surveys amongst cattle practitioners in Bavaria, Germany. The surveys, asking about the occurrence of BNP, were conducted in 2009 and 2010. Answers were analysed spatially by testing for clusters using space-time models. Practitioners were also asked how many cows they serve in their practice and this number was used to estimate the incidence of BNP. Furthermore, in the survey of 2010, practitioners were also asked about usage of vaccine against BVDV.

**Results:**

From the results of the surveys, three clusters were identified in Bavaria. These clusters also coincided with the usage of the specific BVDV vaccine as indicated by the veterinary practices. Furthermore, the representative surveys allow the estimation of the incidence of BNP to be in the order of 4 cases per 10,000 calves at risk.

**Conclusions:**

The study is the only representative survey conducted on BNP. Despite the fact that BNP is a non-infectious disease, regional clusters were identified.

## Background

Beginning in 2006, a rapidly increasing number of calves affected by a haemorrhagic syndrome was noticed within Germany [1] and subsequently also in other European countries, such as Belgium [2], France [3], the UK [4], The Netherlands [5], Italy [6] and others [7–9]. In 2011, the disease was also reported in New Zealand [10, 11]. The disease has been termed ‘bovine neonatal pancytopenia (BNP)’ and was the focus of research in several European countries. A clinical description of the cases has been provided by Friedrich et al. (2019), which is based on cases submitted to the Clinic for Ruminants with Ambulatory and Herd Health Services at the Centre for Clinical Veterinary Medicine, LMU Munich, Oberschleissheim Germany (referred to as ‘clinic’ in the remainder of this paper). Affected calves were mostly less than four weeks of age, and the clinical signs ranged from fever, petechiae in mucosa, cutaneous haemorrhage, and melaena to extensive bleeding of the skin. Affected calves had been born healthy and no clinical signs were obvious until the second or third week of life [1, 12–15]. On post-mortem examination, calves showed generalised haemorrhages and an anaemic appearance. A depletion of cells (panmyelophthisis) was observed in histopathological examinations of the bone marrow, especially in the femur and sternum [1, 2].

In the meantime, the occurrence of BNP has been linked to the use of an inactivated vaccine against bovine viral diarrhoea virus (BVDV) (PregSure®BVD, then Pfizer Animal Health, now Zoetis, Berlin, Germany) [16–22]. In the production of this vaccine, antigens from the permanent bovine kidney cell line, used for the replication of the virus, were contained in the vaccine and caused the production of alloantibodies (antibodies against nonself antigens from members of the same species) in some cows after vaccination [17, 23]. Although many cows vaccinated with this specific BVDV vaccine developed alloreactive antibodies against antigens on bovine cells, dams of affected calves had exceedingly high alloantibody titres [24]. The ingestion of colostrum containing such alloantibodies by neonatal calves was identified as a crucial factor in the pathogenesis of the disease [25, 26]. Almost all calves born to those cows and/or fed with their colostrum developed thrombocytopenia, leucocytopenia and panmyelophthisis, which can vary in degree from subclinical and mild forms to severe and fatal [14, 24, 27, 28]. Due to the thrombocytopenia, affected calves show clinical signs of external and internal haemorrhages such as cutaneous bleeding or blood in the faeces [14, 28]. Now, more than 10 years after the first cases were observed, it is clear that the vaccine is associated with the syndrome; and that additional factors of genetic nature are component causes [29]. It has been proven that these alloantibodies target molecules of the major histocompatibility complex 1 (MHC-I) [20, 24, 27, 30–33]. The MHC-I genes of bovines are highly polymorphic [34, 35]. If the cow has a different allotype than the one contained in the vaccine, she will mount an immune response to this MHC-I antigen and produce alloantibodies. These will then be transferred to her calf via colostrum. If the calf has a similar allotype to the one contained in the vaccine and different from that of its mother, then the alloantibodies ingested with the colostrum will lead to the development of BNP through complement-dependent lysis and/or cytophagocytosis of cells that express the specific MHC-I antigen [27, 32].

Research at the clinic started early on, when affected calves were presented for examination in 2006/2007. Furthermore, even until now, no systematic, representative investigation on the occurrence of the disease exists, neither on the spatial distribution, nor on the incidence of the disease at the herd-level. To address this knowledge gap, the aim of the current study was to describe the spatial pattern of the disease since the first cases had been reported, with a special focus on the time of the early occurrence of BNP in Bavaria, Germany. A further aim was to investigate the incidence of BNP and the usage of vaccines against BVDV by the veterinary practices.

## Results

### First survey of practitioners in July 2009

The time periods covered by the two surveys of veterinary practices coincided with the time when most cases of BNP were observed in Germany (unpublished data of the clinic).

From the questionnaire sent out to large animal practitioners in July 2009, responses were obtained from 456 practices within Bavaria, of which 372 classified themselves as cattle veterinary practices. The others stated that they have ceased practicing or that they only serve horses or small animals. The proportion of practices that completed the questionnaire was 41%.

The number of dairy cows serviced as reported by 283 practitioners ranged from 6 to 12,000. The number of dairy farms serviced as reported by 350 practitioners ranged from 1 to 600. The median number of cows in these practices was 2500 dairy cows (Q1 1030; Q3 4000) on 60 farms (Q1 32; Q3 111).

Of the 372 cattle practices within Bavaria, 91 (24%, 95% confidence interval [CI] 20 to 29%) stated that they had seen or were still seeing suspected cases of BNP.

The practitioners that had seen suspected cases of BNP serviced, on average, a greater number of dairy farms than practitioners that had not observed the disease (median number of dairy farms serviced: 80 versus 60, *p* = 0.050). This in turn also meant, that practitioners that had seen suspected cases of BNP serviced on average more dairy cows than practitioners that had not observed the disease (median number of cows serviced: 3000 versus 2300, *p* = 0.005).

**Fig. 1:**
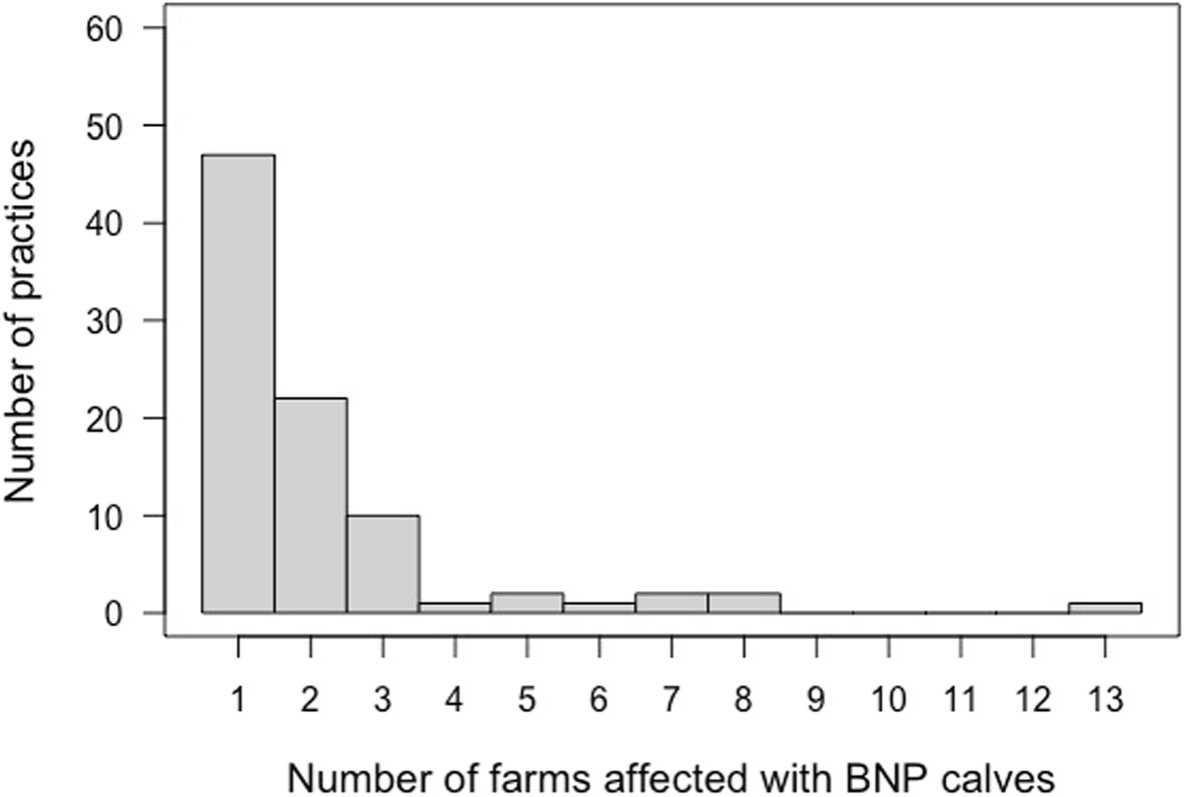
Number of farms affected with BNP-calves within the individual practice areas, as stated by cattle practitioners in Bavaria (*n* = 88) during the first survey of veterinarians in 2009

Of the 91 practitioners that stated that they had seen suspected cases, three did not make any statements about the number of farms or the number of cases they had seen. Of the 88 practitioners providing information on the number of affected farms and cases, just over half reported only one farm (*n* = 47; 53%) while one practitioner reported 13 affected farms within their practice area (Fig. 1).

**Fig. 2:**
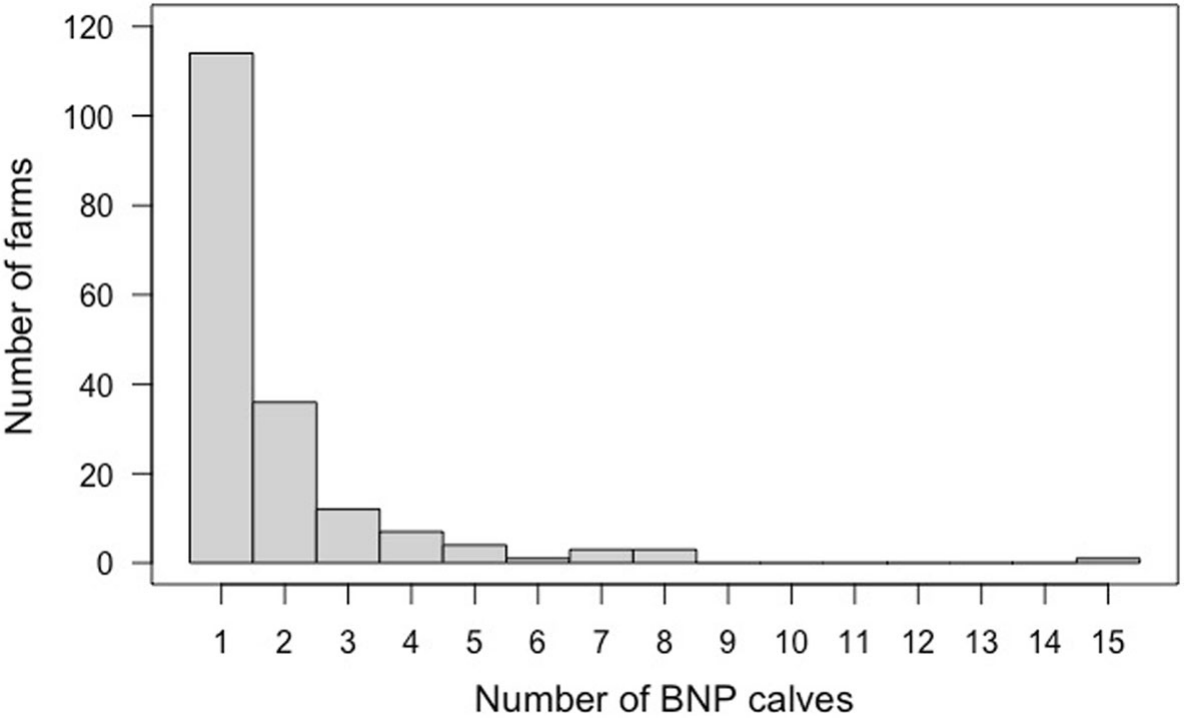
Number of affected calves on each farm as stated by cattle practitioners in Bavaria (*n* = 181 affected farms) during the survey in 2009

The number of affected calves per farm was mostly reported to be a single calf (*n* = 114; 63% of all affected farms), followed by two calves (*n* = 36; 20% of all affected farms); for one farm 15 cases were reported (Fig. 2).

**Fig. 3:**
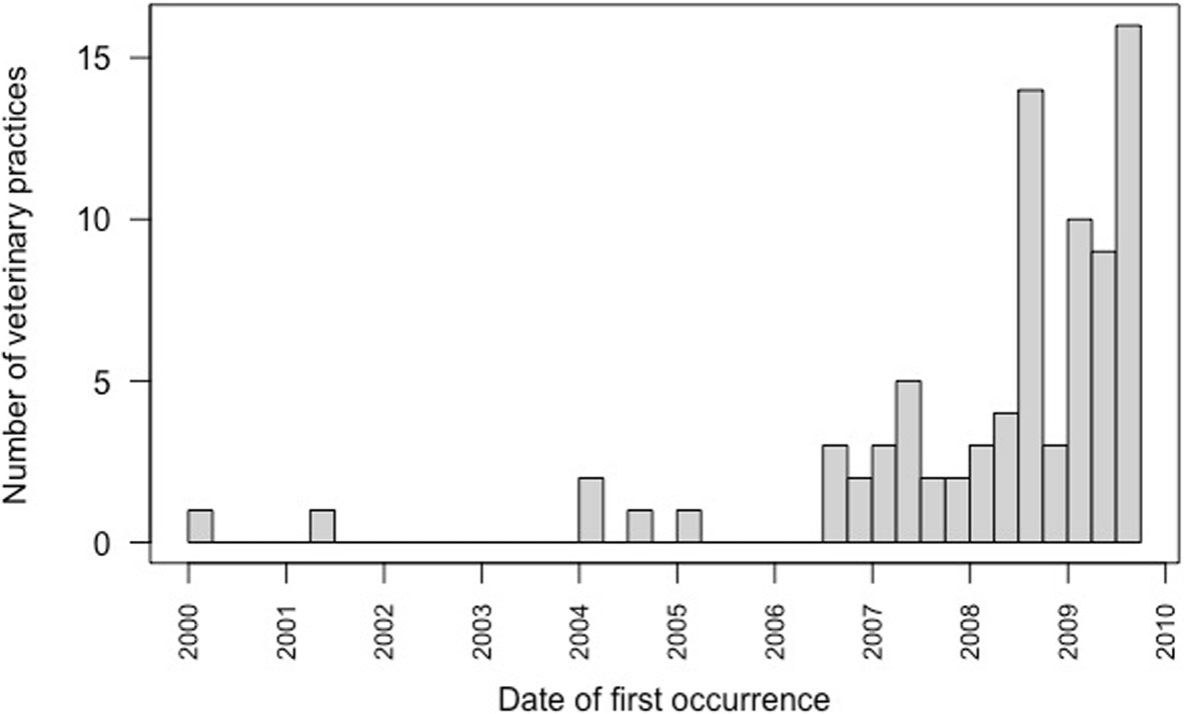
Date when BNP cases were first seen as stated by veterinary practitioners (*n* = 82) in Bavaria during the survey of 2009

Of the 91 veterinary practices in Bavaria with BNP cases, 82 respondents provided a date on which they had first seen the disease. Thirty-two of these (39%) stated that they had seen the disease over a period of more than one year. While the earliest first observation date was in 2000, most cases were seen from 2006 onwards (Fig. 3).

### Second survey of practitioners in July 2010

In the second survey, 197 veterinary practices from within Bavaria responded (19% return of 1058 practicing veterinarians). Of these, 156 had already taken part in the previous survey, while 41 practices answered in 2010, but not in 2009. In total 56 of the 197 practitioners (28%; 95% CI 22 to 35%) reported that they had seen BNP-affected calves amongst the cattle of their clients.

The practices in which the practitioners had observed BNP serviced a median of 80 dairy farms (reported by 51 practitioners) and 3000 dairy cows (reported by 42 practitioners), while the practices in which the practitioners had not observed BNP serviced a median of 64 dairy farms (reported by 139 practitioners) and 2500 dairy cows (reported by 125 practitioners). The number of dairy farms and the number of dairy cows being serviced by the practices was higher in practices that had observed BNP versus practices that had not observed cases (*p* = 0.043 for the number of farms and *p* = 0.022 for the number of cows respectively).

The years when the disease was seen and the estimated number of calves observed during these years, were stated by 50 practitioners and are summarised by year in Table 1. Most cases were seen in 2009, although in 2010 until August, when the survey took place, there were already 74 cases observed.

**Table 1:** Number of BNP calves observed as stated by practitioners in Bavaria during a survey in August 2010

Year	2005	2006	2007	2008	2009	Until July 2010
Number of BNP calves	1	3	1	76	128	74

The practitioners were also asked to provide the total number of farms that had been affected and the number of calves affected in those farms over the whole period from the first occurrence until August 2010. Fifty of the 56 practitioners that had seen BNP replied to this question and stated between one and seven farms, where they had observed BNP calves. In total, 112 farms had BNP calves. These practices serviced in total around 5354 dairy farms. This returned an incidence risk of BNP on farm level of 2 (95% CI 1.7 to 2.5) BNP-positive farms per 100 farms. The number of affected calves on each farm was provided for 107 farms. On most farms, only one or two calves were affected (*n* = 86, 77%), while on one farm 15 calves and on two others eight calves on each of these two farms were affected.

Practitioners were also asked to provide the number of cows on the reported farms with BNP calves. For 55 of the 112 farms this was provided and in total these 55 farms had 2972 cows (an average of 54 cows per farm). Assuming one birth per cow each year the estimated number of calvings on these 55 farms over a period of 4.5 years (that is, from 2006 until mid-2010, the time when most BNP cases occurred) was 13,374. On these 55 farms a total of 94 BNP calves were reported by the practitioners, which leads to a calf-level incidence risk of BNP on calf-level of 7 (95% CI 5.7 to 9.6) BNP cases per 1000 calving events.

For estimating the incidence risk over all farms, the number of reported BNP-calves was divided by the estimated total number of dairy cows serviced by the practices over a period of 4.5 years. In total, 167 practices stated the number of dairy cows they are servicing within their practice, which summed up to 505,900 dairy cows in total. In total, 213 BNP-calves were reported by 42 of these practices. The calf-level incidence risk was 4.0 (95% CI 3.7 to 4.8) BNP cases per 10,000 calving events.

For estimating the incidence risk of BNP at the farm level, the number of affected farms (*n* = 112) was divided by the total number of dairy farms serviced by all the practices that replied to the survey (a total of 16,845 dairy farms), which yielded an estimated incidence risk of 0.7% (95% CI 0.5 to 0.8) BNP-positive farms per 100 farms during the 4.5 years covered by the survey.

Of the 197 practitioners that replied in the second survey, 124 provided information about the vaccines against bovine viral diarrhoea (BVD) they had been using at the time of the questionnaire being administered, whereby they could state multiple ones. In total 186 declarations of the use of BVDV vaccines were provided (Table 2), of which the vaccine Bovidec® (Virbac, Bad Oldesloe, Germany) was reported most frequently, followed by Bovilis®BVD (Intervet, MSD, Unterschleissheim, Germany), Vacoviron® (then Merial, now Boehringer Ingelheim, Ingelheim, Germany) and PregSure®BVD (then Pfizer Animal Health, now Zoetis, Berlin, Germany). Forty-two of these 124 veterinary practices (34%) were amongst those that had observed BNP-calves. The specifications ot the vaccine brands used are provided in Table 2.

**Table 2:** Vaccines being used against bovine viral diarrhoea virus (BVDV) at the time of the survey and the number of times these vaccines were mentioned by practitioners (*n* = 124) in a survey of BNP in 2010

Vaccine Name	Number times mentioned in total	Number times mentioned by 42 veterinary practices with observed BNP cases	Number times mentioned by 82 veterinary practices without observed BNP cases
Bovidec® (Virbac)	52 (28%)	13 (19%)	39 (33%)
Bovilis®BVD (Intervet)	36 (19%)	12 (17%)	24 (21%)
Vacoviron® (Merial)	35 (19%)	10 (15%)	25 (21%)
PregSure®BVD (Pfizer)	34 (18%)	25 (36%)	9 (8%)
Mucobovin® (Merial)	26 (14%)	7 (10%)	19 (16%)
Rispoval 3-BRSV-PI3-BVD® (Pfizer)	3 (2%)	2 (3%)	1 (1%)
Total number of declarations	186 (100%)	69 (100%)	117 (100%)

The distribution of vaccine usage was different between veterinary practices that stated they had never seen BNP versus such practices that had seen BNP (*p* < 0.001), whereby the practices that had never seen BNP cases used mostly Bovidec® (Virbac, Bad Oldesloe Germany), while the practices that had seen BNP cases mostly used PregSure®BVD (Zoetis, Berlin, Germany). In total, 25 of the 42 veterinary practices that answered this question and that had seen BNP cases used the specific BVDV vaccine (PregSure®BVD, Zoetis, Berlin, Germany), while only nine of the 82 practices that had never seen BNP used this vaccine (p < 0.001). Those practices that used PregSure®BVD (*n* = 34) were 3.9 (95%CI 2.4 to 6.3) times as likely to have seen BNP in their practice compared with those practices that used other BVD vaccines.

### Spatial distribution of the replies to the surveys 2009 and 2010

**Fig. 4:**
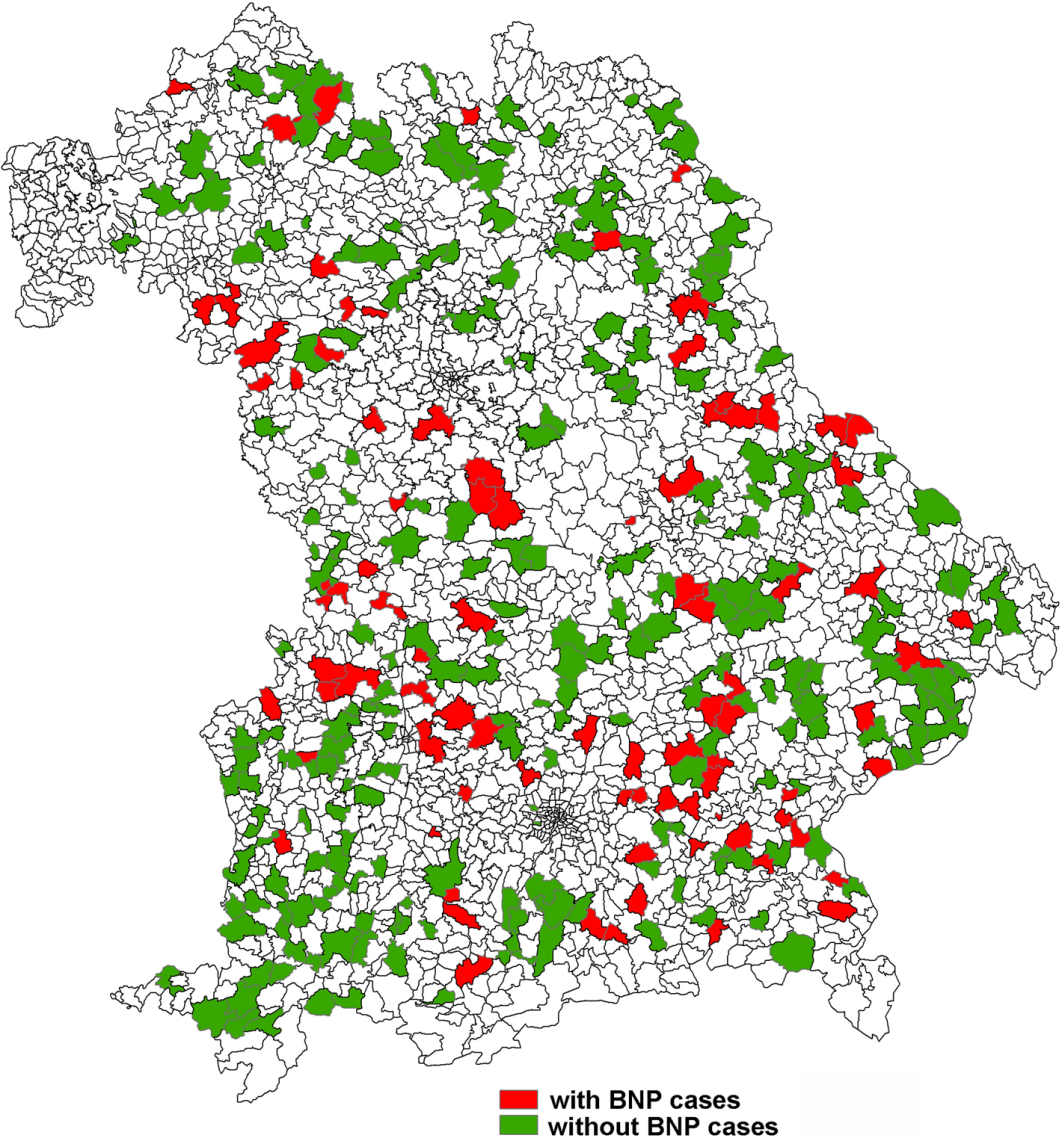
Geographical distribution of the postal zip codes with cattle practices that have replied in the survey, and whether they had observed cases of BNP or not, as stated during the survey of 2009 (*n* = 368). Map depicted here was generated as part of the current study

The geographical distribution of the veterinary practices that responded to the questionnaire in 2009 and their answers to whether they had observed BNP in their practice or not, is provided in Fig. 4. For four of the 372 respondents, the postal zip code was not given and could not be matched to a veterinary practice.

**Fig. 5:**
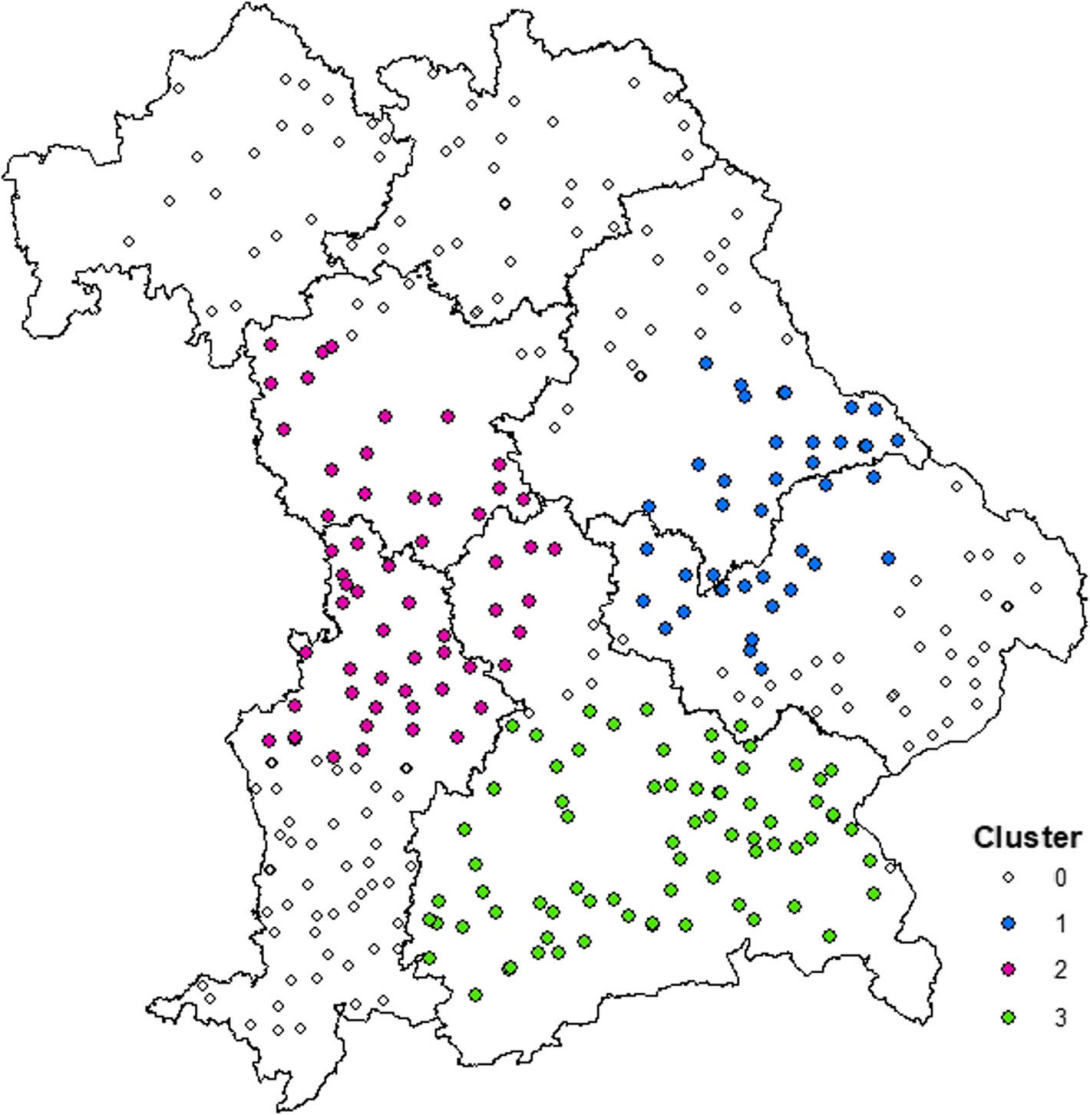
Spatial clusters as identified by SaTScan of veterinary practices during a survey in 2009 on the occurrence of BNP cases (368 veterinary practices; 91 with BNP-cases; three clusters identified). Map depicted here was generated as part of the current study

Using Kulldorff’s space-scan statistic, no purely temporal or purely spatial clusters were identified, but three space-time clusters (Fig. 5) were identified using the maximum window size of 25% in time and space: one of them in the East of Bavaria (with a relative risk [RR] of 4.51, *p* < 0.001, radius of 72 km), the second in the West of Bavaria (RR = 3.81, p < 0.001, radius of 51 km) and the third in South Bavaria (RR = 3.20, *p* = 0.004, radius of 73 km). Each of the clusters consisted of cases occurring in 2008 and 2009. Using a maximum cluster size of 10% in space and time, only the first two clusters were identified, while the cluster in the south was not statistically significant. The first cluster was in the same area as the west-cluster using 25% window size and comprised cases occurring in 2008 (RR = 4.30, p = 0.004) and the second cluster was in the same area as the east-cluster using 25% window size, comprising cases occurring in 2009 (RR = 4.30, p = 0,004).

**Fig. 6:**
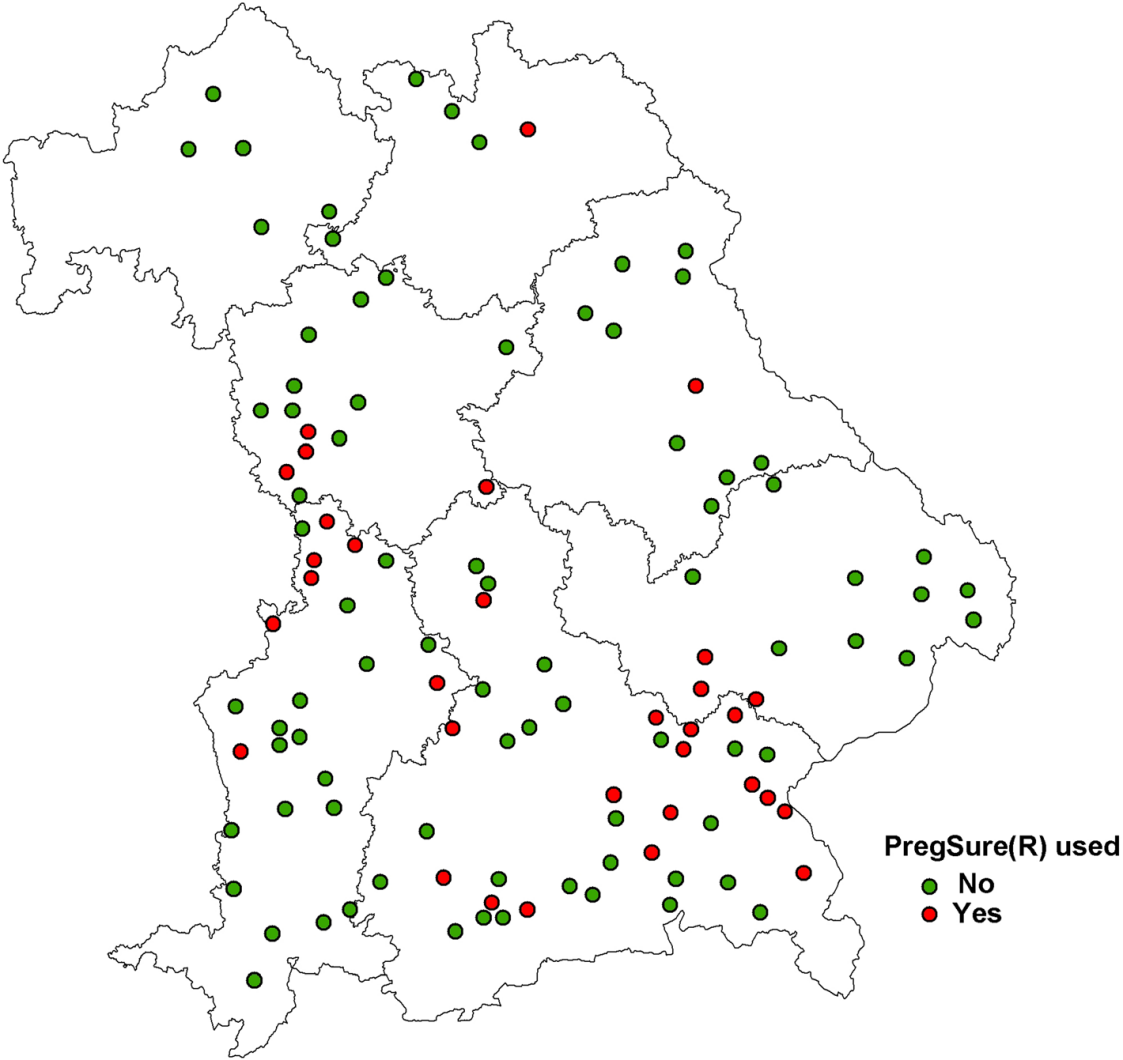
Spatial distribution of practices responding to a questionnaire in 2010 in relation to the information whether they used PregSure®BVD or not. Map depicted here was generated as part of the current study

Plotting the 124 practices that had provided information on the BVD vaccines used, these clusters are also visible in Fig. 6, which displays the information whether practices have used PregSure®BVD or not.

## Discussion

Bovine neonatal pancytopenia is a disease that was first described in 2009 [1]. The first case of BNP was referred to the Clinic for Ruminants with Ambulatory and Herd Health Services at the Centre for Clinical Veterinary Medicine, LMU Munich, Oberschleissheim Germany in 2006 [1, 36]. Since then, the number of cases increased continuously with a peak in case numbers in 2009. Thereafter, more and more information became available, such as the potential involvement of a specific BVDV vaccine (PregSure®BVD, Zoetis, Berlin, Germany) and the involvement of colostrum in the development of the disease. Thus, many farmers that had affected calves in the previous years changed their colostrum management and/or the BVD vaccine. These changes led to a decrease in observed and reported cases. It is now well known that the depletion of leucocytes and thrombocytes is caused by maternal antibodies transferred to the calves through colostrum [25, 26, 32]. These specific maternal alloantibodies are produced in reaction to the vaccination with an inactivated vaccine (PregSure®BVD) [24, 26, 27, 32]. However, the role of the specific BVDV vaccine (PregSure®BVD) was long debated because the disease was not spatially evenly distributed and because there was no association with the timing of the vaccination. In Germany, most cases occurred in Bavaria and fewer cases in the other federal states, which was later linked to different vaccination schemes supported by the federal animal disease fund [20]. Nevertheless, even on a smaller scale within the federal states and within other countries, regional differences were observed for which the reasons were not known [37].

BNP is not notifiable in Germany in a sense that cases have to be reported like cases of BVD itself [38]. However, practitioners and farmers were asked to report the suspect cases, with or without confirmation, to the German Pharmacovigilance system at the Paul-Ehrlich-Institute. Furthermore, as the reports were voluntary reports by farmers and practitioners, no representative data have been available on the spatial distribution of BNP cases within Germany. To the best of our knowledge, the surveys presented here are the only representative investigations on the spatial distribution in any affected country.

BNP is a disease with very low incidence but a very high case fatality rate (more correctly termed a case fatality risk in the context of this study). The low morbidity was also seen in the surveys conducted with practitioners. Practitioners were coordinating their answers amongst their colleagues within the practice and provided one form per practice. About a quarter of the practitioners that answered the questionnaire stated that they had seen the disease within their practice. Most of them stated only one to two farms being affected by the disease, although they serviced a median number of 65 dairy farms. Affected farms mostly had only one animal affected. If a disease is non-infectious, but just occurs sporadically, then a practice that services more farms has a higher chance to see the disease than a practice with a lower number of dairy farms being serviced. However, as it is more likely that a veterinary practice uses only one brand of vaccine, it would be expected that several farms were affected and several cases occur per practice. Most practitioners noticed the disease in 2008/2009. However, one practice stated that they had seen the disease as early as 2000. The earliest cases of BNP reported in the literature were from 2006 [13]. Retrospectively, it cannot be clarified if the signs observed in 2000 were unrelated to BNP [39]. Alternatively, this could have been a case of haemorrhagic diathesis of unrelated aetiology, as seen in other studies [16, 22]. Such idiopathic cases have been described by Stoll et al. (2016) prior to the first description of PregSure®BVD related BNP. It can also not be excluded, that other cases of haemorrhages seen by the practitioners were classified as BNP-cases [39]. Additionally, recall bias cannot be ruled out, in that practitioners did not remember the details of cases that had occurred many years before. However, the clinical signs in BNP are very distinct and considered dramatic by farmers and practitioners, so for this reason it is unlikely that practitioners did not remember such cases.

The results of the surveys reported in this paper allow the estimation of the farm-level and individual animal level incidence risk of BNP. Due to the fact that the disease is not notifiable, the incidence risk of the disease was estimated so far via different methods, such as the number of cases per doses of PregSure®BVD sold, which was found to be 10 BNP cases per 10,000 PregSure®BVD doses sold in Bavaria [20]. Assuming that most of the time at least three doses were needed (basic vaccination and one booster vaccination) in order to provoke BNP (K. Cussler, pers. comm.) this would result in an incidence risk of a minimum of three BNP cases per 1000 vaccinated cows. This is in agreement with the results of the present study, where seven BNP cases per 1000 calvings were found on farms that were affected by BNP and thus, most likely had their cows vaccinated with PregSure®BVD.

In the present study, about a quarter of the veterinary practices had seen the disease until mid 2010 when the second survey was conducted. At the farm-level, the estimated farm-level incidence risk in this study was 0.7%, which agrees with the impression that the disease only shows a low morbidity over the whole cattle population.

At the individual animal level over the whole cattle population an incidence risk of four BNP-positive calves per 10,000 calvings was estimated, using the number of observed cases and the number of dairy cows being serviced in the practices. It is not possible to verify this number. Although several institutes, amongst them also the PEI (Paul Ehrlich Institute) and universities were collecting data, there is no combined information available. However, until February 2011, there were already more than 3000 cases of BNP in Germany and more than 4500 in Europe [40]. In total, there were about 6000 calves reported to the Paul-Ehrlich-Institute over all the years. With approximately 4.5 million dairy cows in Germany this yields an incidence risk at the individual animal level of 2.9 BNP cases per 10,000 calvings over the time period of 4.5 years. This number however, is a conservative estimate, as there is a high proportion of underreporting likely due to the fact that there is no obligation for notifying the disease and no compensation was paid for affected calves. Reichmann et al. (2016) found a high proportion of underreporting in their study comparing cases reported to the clinic versus the official pharmacovigilance system. The estimated individual animal incidence risk found in this study was therefore greater than the 2.9 BNP cases estimated through the official numbers. It is expected that dairy farmers with affected calves mentioned these events to their veterinary practitioners more likely than submitting an official written report to the authorities. Nevertheless, it is possible that this incidence risk is underestimated as well, as the practices might not have known of all affected farms and/or animals and because some farms sell their male calves at a very young age, possibly before they would have developed BNP. We conclude that the incidence risk of BNP is likely to be higher than that estimated in this study.

Although the collection of information of cases provides some information about regional differences, it does not give a representative picture of the actual disease distribution. To this end the surveys were conducted in 2009 and 2010. We are not able to deduct from this the exact geographical location of the farms with BNP-cases. However, traditionally veterinary practice clientele are usually in close proximity to veterinary practices in Bavaria.

It is not possible to calculate the exact proportion of returns, as the veterinarians registered in Germany in the veterinary chamber are individuals and are not matched with veterinary practices. Often several veterinarians work in one veterinary practice and faxes were received from the practice not from the individual veterinarians. Thus, the apparent return rate was 41%, which is considered very high. We are very confident that the results obtained in this study are sufficiently close to representative of the BNP situation in Bavaria. Due to the short questionnaire, equal response-rates between practitioners that had observed the disease and those that had not observed the disease are assumed. This cannot be ensured. In the second survey (2010), the apparent return rate was lower with 19%, which could have been due to different reasons. It could be that the awareness was not as high in 2010 as it had been in 2009, as the disease was not as present in the media then as it was in 2009, or that practitioners did not have the same interest in the disease as in the previous year. Interestingly, the percentage of practices that observed the disease was similar in both surveys (25 and 28%).

Most responding practices in the survey provided their postal zip code allowing the geographical location of their practice to be estimated. The geographical distribution of the affected practices in this study showed three distinct clusters. The areas of the clusters found in the regional distribution of the practices affected by BNP coincide with the areas, where the practitioners used the vaccine PregSure®BVD. However, on the other side, it cannot be excluded that some of the veterinary practitioners, which observed the disease in 2009, already had changed their BVD-vaccines in 2010, thus underestimating the usage of PregSure®BVD. This means, there is a spatial association of using PregSure®BVD and the occurrence of BNP, which confirms once more the association between the vaccine usage and the occurrence of BNP. Seventy-three practices did not provide any information on vaccines they used for vaccinating against BVD, which could mean they did not vaccinate against BVD or they did not give the information. We could not evaluate this and therefore, these practices were excluded from this part of the analysis. The fact that spatial clusters existed in the distribution of the affected practices seems to contradict the assumption that the disease is non-infectious. However, it can be hypothesised that veterinary practices used one specific BVD-vaccine predominantly on all of their client’s farms. Therefore, it is conceivable, that within the practices the occurrence of BNP clustered. It is also conceivable that neighbouring practices were influencing each other, or were influenced by the same representative of the pharmaceutical company, giving rise to geographical clustering.

During the time of the surveys (2009 and 2010) the association between the BVD-vaccine PregSure®BVD and BNP was only hypothesised and studies were underway to investigate different risk factors. PregSure®BVD was brought onto the market at the end of 2004 and rapidly gained a high market share in Germany (personal communication by representative of Pfizer, 2010). Other European countries followed in licensing PregSure®BVD during 2005. For induction of a good immunity against BVD, the manufacturer recommends a basic immunisation with two vaccinations within a short time period (4 to 8 weeks), with annual booster vaccinations. In the current study, PregSure®BVD was only stated in 18% of the cases, where BVD vaccines were named by the 124 practitioners. However, it is likely that some of the practitioners already changed their BVD-vaccine by 2010, when we asked this question. Furthermore, from the information of the surveys we are not able to deduct the proportion of cows being vaccinated with PregSure®BVD, as practitioners could mention several BVD-vaccines they used.

Now, more than 10 years after the first case was observed, it is established that the vaccine is definitely part of the cause of the syndrome; and that additional factors of genetic nature are component causes [29]. The occurrence of BNP and its interrelation to a licensed vaccine shows clearly the implications for the production of veterinary vaccines on homologous cell lines.

## Conclusion

This is the first study that describes a representative distribution of BNP cases. Due to the fact that BNP is not notifiable in Germany, there is no complete picture about the spatial distribution available. Therefore, the present study of two representative surveys of cattle practitioners within Bavaria was the only study to examine the regional distribution of the disease. Although the disease is non-infectious, but autoimmune-related, the disease was not randomly distributed, but showed clustering.

## Methods

The extent to which BNP occurred in the cattle population of Bavaria in the first years after its occurrence was the focus of this study.

### Surveys of practitioners in Bavaria during the years 2009 and 2010

Information on the occurrence of BNP observed by cattle practitioners in Bavaria was collected through a questionnaire, which was sent out in July 2009 to all large animal practitioners in Bavaria, Germany (through the Veterinary Chamber of Bavaria, *n* = 1124; according to the law § 1 ‘Meldeordnung’ each veterinarian is required to register with the District Veterinary Association of the federal state, where he/she is practicing or living). Additionally, the survey was published in the “Deutsches Tierärzteblatt” (the journal of the German Veterinary Association, which is sent to each veterinarian within Germany) to remind practitioners about the survey and to increase the proportion of returns. Practitioners from outside of Bavaria were also able to respond, however, their answers (*n* = 65) were not included in the analyses presented in this study.

To keep the questionnaire short and concise two pages were sent out (see Additional file 1). The first page contained a short description of the disease and the request to fill in the second page, which contained the questionnaire consisting of four questions. It was asked whether the practitioners had seen the disease in the farms they serviced or not, irrespectively of the fact that the disease was confirmed or not. Additionally, they were asked to provide the number of dairy cows and farms they serve. They were also asked about the year, when they had seen the first of such cases. In the final question they were asked to state the number of affected farms and suspected cases on these farms in total. Practitioners were asked to state the name of the farm or the first two letters of the affected farms only. Information was entered only with the first two letters to ensure anonymity of the information provided. The four questions were designed on the basis of minimal information required to investigate the spatial distribution of BNP. A facsimile number of the clinic was provided, where the practitioners were asked to send their filled-in questionnaires to. Practitioners could also send the questionnaire by mail or by email after scanning. The practitioners were also asked to coordinate the answers with their colleagues, if they worked in a veterinary practice with more than one bovine practitioner. In addition, on the form they were asked to provide the name of the practice. Using the practice name, we could ensure that information was only provided once per practice. Thus, the information used for the current study is at the level of veterinary practice. However, as practitioners have replied to the questionnaire on behalf of their practice, we will refer to the responses by the term ‘practitioners’. The facsimiles received from the practitioners were numbered sequentially and only this number was entered into the database, no personal information of the practitioners was entered, thus providing anonymity.

All cattle veterinarians in Bavaria (*n* = 1058) were contacted again one year later, during August 2010, asking the same information as in 2009, plus additional information on vaccines used in their practice (see Additional file 2). As indications towards an association between a specific vaccine against BVDV and BNP were increasing in 2009, this second survey was undertaken to ask specific information on vaccinations against BVDV from practices within Bavaria.

### Statistical analyses

The proportion of veterinary practices that had seen the disease was calculated. Clopper-and Pearson 95%-confidence intervals were calculated for this proportion. The number of farms and dairy cows within the practices were compared between practices on the basis whether they had observed the disease or not, using Mann-Whitney-U-tests. Calculations were performed and figures produced using R [41].

In calculating the incidence estimates, the number of reported cases of BNP per 1000 or 10,000 calving events are stated. For practical purposes this is equivalent to (but not exactly the same as) the number of cases of BNP per 1000 or 10,000 calves, respectively.

Geographical analyses were conducted with ArcMap (version 9.3; www. esri.com) and ArcGIS (version 10.5; www.esri.com). The distribution of the practices, which had responded to the fax surveys in 2009 and 2010 were shown using the polygons of the postal zip codes of the practices. If two practices were registered under the same postal zip code, and one of them had observed BNP-cases and the other had not, then this postal zip code polygon was depicted on the map as having observed BNP cases. For identifying space-time clusters, the spatial scan statistic was used [42]. For this analysis, the coordinates of the centroid of each postal zip code was used as a proxy for the location of each practice. If two or more practices were registered under the same postal zip code, the coordinates were altered slightly to obtain individual sets of coordinates within that same postal zip code polygon. The alteration was done manually in randomly selecting a point within the same postal zip code polygon. The Bernoulli model within SaTScan was selected because the practices had either observed the disease or not [42]. The time resolution for the space-time-scan statistic was set to one year. For the cases, the year when BNP-cases were observed for the first time was selected, while for the controls, a year between 2000 and 2009 was chosen at random. The Kulldorff space-time-scan statistic is calculated by using a circular window, which is then moved in space and time. For each circular window, the null hypothesis is tested against the alternative hypothesis, that there is an elevated risk of BNP cases occurring within a window compared to outside the window. For each window the maximum likelihood ratio is calculated. The maximum cluster size was varied between 10 and 25% of the population (both for time and space). A cluster was determined to be statistically significant if the *p*-value was less than 0.05.

## Supplementary information

**Additional file 1.** Table 1 Fax questionnaire sent out in 2009

**Additional file 2.** Table 2. Fax questionnaire sent out in 2010

## Data Availability

The datasets used and analysed for this study are available from the corresponding author on reasonable request.

## Abbreviations

BNP: bovine neonatal pancytopenia
BVD: bovine viral diarrhoea
BVDV: bovine viral diarrhoea virus
PEI: Paul-Ehrlich-Institute
