# Supplementary material for: Spatial distribution and incidence of bovine neonatal pancytopenia in Bavaria, Germany
Source: BMC Vet Res. 2020 May 24;16:155. doi: 10.1186/s12917-020-02371-x (PMC7245873; doi:10.1186/s12917-020-02371-x)
Supplement: Supplementary file 1 — Additional file 1. Table 1 Fax questionnaire sent out in 2009 [file 12917_2020_2371_MOESM1_ESM.pdf]

Questionnaire sent out to cattle practitioners in 2009

Questions:

1. How many dairy farms / dairy cows are you serving in your practice?

   ____
2. Have you observed this disease in ‚your' farms?
   O no (if this is the case, please send us your answer nevertheless)
   O yes
3. If yes:
   In which timeframe: from (Month/Year) to (Month/Year)
   ________/_________ to __________ / ________

Farm (anonymised)	Number of affected calves	Number of cows on the farm

If there are not enough rows in the table, please add in a separate page.

PRACTICE: ____

Tel. Nr. ____________________________ Fax. Nr. _________________________

In case you answered with yes, we might ring you back and ask for more information.

Please send your answer back by fax or mail to:

Clinic for Ruminants
Sonnenstr. 16
85764 Oberschleissheim

FAX Number:

For further information:

Dr.
Dr.
Tel:

THANK YOU VERY MUCH FOR YOUR SUPPORT!
