# Supplementary material for: Spatial distribution and incidence of bovine neonatal pancytopenia in Bavaria, Germany
Source: BMC Vet Res. 2020 May 24;16:155. doi: 10.1186/s12917-020-02371-x (PMC7245873; doi:10.1186/s12917-020-02371-x)
Supplement: Supplementary file 2 — Additional file 2. Table 2. Fax questionnaire sent out in 2010 [file 12917_2020_2371_MOESM2_ESM.pdf]

Questionnaire sent out to cattle practitioners in 2010

Questions:

1. How many dairy farms / dairy cows are you serving in your practice?

   ____

2. Have you observed this disease in ‚your' farms?
   O no, not until last year, and now not either (if this is the case, please send us your answer nevertheless)
   O yes, haven't observed it until last year, but since then
   O yes (if you have answered last year, we only need the information of the new incident cases)
3. If yes:
   In which timeframe: from (Month/Year) to (Month/Year)
   _________/_________ to __________ / ________
4. In which years did you observe how many calves?

Year	2005	2006	2007	2008	2009	2010
Number of calves						

5. Number of farms and number of affected calves

Farm (anonymised)	Number of affected calves	Number of cows on the farm

6. Which vaccination scheme are you using in the farms, where you vaccinate against BVD?
   Which vaccine? ____________________________
   Base vaccination? O yes O no
   Yearly vaccination? O yes O no
   Have you changed the vaccination scheme during the last year? O yes O no
   If yes, in what way?

Please fax or send this page back to:
Clinic for Ruminants, Sonnenstr. 16, 85764 Oberschleißheim
FAX:

If you have questions or comments, please call us:
Tel:

Practice stamp:

THANK YOU VERY MUCH FOR YOUR SUPPORT!
